# Patient Satisfaction with Healthcare Services and the Techniques Used for its Assessment: A Systematic Literature Review and a Bibliometric Analysis

**DOI:** 10.3390/healthcare11050639

**Published:** 2023-02-21

**Authors:** Diogo Cunha Ferreira, Inês Vieira, Maria Isabel Pedro, Paulo Caldas, Miguel Varela

**Affiliations:** 1 Centre for Public Administration and Public Policies, Institute of Social and Political Sciences, Universidade de Lisboa, Rua Almerindo Lessa, 1300-663 Lisbon, Portugal; 2 CERIS, Instituto Superior Técnico, University of Lisbon, Av. Rovisco Pais 1, 1049-001 Lisbon, Portugal; 3 Instituto Superior Técnico, University of Lisbon, Av. Rovisco Pais 1, 1049-001 Lisbon, Portugal; 4 CEGIST, Instituto Superior Técnico, University of Lisbon, Av. Rovisco Pais 1, 1049-001 Lisbon, Portugal; 5 Business and Economic School, Instituto Superior de Gestão, Av. Mal. Craveiro Lopes 2A, 1700-284 Lisbon, Portugal; 6 Centre for Local Government, UNE School of Business, University of New England, Armidale, NSW 2350, Australia; 7 CEFAGE, Faculdade de Economia, Universidade do Algarve, Campus of Gambelas, 8005-139 Faro, Portugal

**Keywords:** patient satisfaction, health services, quality management, systematic review, bibliometric analysis

## Abstract

Patient satisfaction with healthcare provision services and the factors influencing it are be-coming the main focus of many scientific studies. Assuring the quality of the provided services is essential for the fulfillment of patients’ expectations and needs. Thus, this systematic review seeks to find the determinants of patient satisfaction in a global setting. We perform an analysis to evaluate the collected literature and to fulfill the literature gap of bibliometric analysis within this theme. This review follows the Preferred Reporting Items for Systematic Reviews and Meta-Analysis (PRISMA) approach. We conducted our database search in Scopus, Web of Science, and PubMed in June 2022. Studies from 2000–2021 that followed the inclusion and exclusion criteria and that were written in English were included in the sample. We ended up with 157 articles to review. A co-citation and bibliographic coupling analysis were employed to find the most relevant sources, authors, and documents. We divided the factors influencing patient satisfaction into criteria and explanatory variables. Medical care, communication with the patient, and patient’s age are among the most critical factors for researchers. The bibliometric analysis revealed the countries, institutions, documents, authors, and sources most productive and significant in patient satisfaction.

## 1. Introduction

Healthcare systems are continually changing and improving, and so it is necessary to find a way to assess outputs while evaluating the satisfaction of the service receiver, in this case, the patient. One can define patient satisfaction as a patient’s reaction to several aspects of their service experience. Assessing patient satisfaction may provide valuable and unique insights about daily hospital care and quality. One widely accepts it as an independent dimension of care quality that includes internal aspects of hospital care. Patient satisfaction is a concept that has long been neglected and cast aside, but is becoming gradually more important. Donabedian [1] includes it as an outcome of healthcare services; hence, it is of utmost importance to evaluate care quality. Several authors argue that satisfaction and the result in terms of the patient’s health status are related terms [2,3,4,5]. Thus, the present study sheds light on the factors that most influence patient satisfaction. With this information, managers can more efficiently allocate resources to improve patients’ experience and satisfaction [6].

Measuring healthcare quality and satisfaction constitutes an indispensable element for adequate resource management and allows for the focus on its users’ preferences, giving them a chance to construct a customized health service, better fitted to their needs and expectations [7]. When talking about public hospitals, there may not be a financial interest in performing these studies since they are not particularly interested in profit. However, with the increase in market competitiveness, private companies need to meet patients’ needs, satisfying them so that they then become loyal to the organization [8]. Patient satisfaction can be useful for structuring evaluations referring to patient judgments according to inpatient care. It is relevant from an organizational management perspective [9]. Patient satisfaction and quality of health services are, thus, crucial elements for the long-term success of health institutions [10].

Despite the high number of studies regarding this topic, the results are inconclusive and differ across each document [11,12,13]. Contradicting evidence exists across patient satisfaction studies due to its subjective nature [14]. Since each individual has his/her perceptions, satisfaction is nothing but a relative concept, influenced by individual expectations and evaluations of health services’ attributes [15].

Several systematic reviews have analyzed the determinants of patient satisfaction: Naidu [16], Almeida et al. [17], Farzianpour et al. [18], and Batbaatar et al. [14] are some noteworthy recent reviews. Similarly, reviews on the methods most utilized by researchers are scarce, and none of them delivers a comprehensive and profound analysis of literature through bibliometric tools. Thus, this analysis aims to assess the different aspects of patient satisfaction in a global healthcare context, along with the identification of the main countries, institutions, documents, authors, and journals of this research area, with co-citation and bibliographic coupling networks. This systematic review can contribute to the knowledge of patient satisfaction, whether the influential factors, or the most advised methodology, and be an essential input for researchers or scholars interested in the study of patient satisfaction. In addition, we conducted a meta-analysis by statistically analyzing the main factors underlying patient satisfaction and the main methodologies adopted for its study.

The incessant demand for improved results and quality of health services offered is of extreme importance in developing a more effective organizational policy adjusted to the patients’ needs. Health organizations recognize that service quality is especially pertinent regarding the healthcare market’s promotion and public image [19]. Hence, patient satisfaction surfaces as a variable for promoting health organizations’ quality, allowing an assessment and identification of patients’ most relevant dimensions and their satisfaction level. Patient satisfaction helps to measure the quality of healthcare, thus becoming an essential and frequently used indicator. It affects clinical outcomes, medical malpractice claims, and timely, efficient, and patient-centered healthcare delivery [20]. Patient satisfaction and quality of health services are a priority for the services industry due to increasing consumption and are critical elements for health institutions’ long-term success [10,21].

Even though satisfaction is an essential aspect of quality, the relationship between these two concepts is not linear. On the one hand, satisfaction studies’ results can be ambiguous and may not always be impartial. Given that patients evaluate physicians’ performance, most missing the necessary abilities, results can be based on affinity and not on the health professional’s technical skills. On the other hand, providers may have to face a trade-off between providing satisfaction to their patients or better treatment outcomes [22]. Since each person has his/her perceptions, satisfaction is nothing but a relative concept, influenced by individual expectations and evaluations of health services’ attributes [15]. Patient satisfaction is complex to assess, given its multidimensionality. It is composed of diverse aspects that may not be related to the patient’s service’s actual quality.

It is valuable to consider the highly cited Donabedian framework on how to examine health services’ quality in order to surpass the current lack of clarity on defining and measuring satisfaction, and to evaluate the quality of medical care using three components [1,23,24,25]—structure, process, and outcome (results):

- Structure: Environment, provider’s skills, and administrative systems where healthcare occurs;
- Process: The constituents of the received care (measures doctors and medical staff considered to deliver proper service); and
- Outcome: The result of the care provided, such as recovery, avoidable readmission, and survival;

The conceptualization of patient satisfaction regarding expectations and perceptions is related to Donabedian’s triad. For instance, the patient will be satisfied with hospital attributes if his/her expectations are met [25]. However, one of the leading criticisms of patient satisfaction ratings is the incapacity to rationalize medical care expectations, which can be affected by previous healthcare experiences [26]. The same happens with the other two components. The patient will be satisfied with the process if symptoms are reduced. The outcome will be favorable if there is a recovery, demonstrating that received care perception meets prior expectations. Throughout his framework, Donabedian regarded “outcome” as the most crucial aspect, defining it as a change in a patient’s current and future health status that can be confidently attributed to antecedent care [22].

## 2. Materials and Methods: Data Collection and Extraction Method

We performed this research respecting the guidelines of the Preferred Reporting Items for Systematic Reviews and Meta-Analysis (PRISMA) statement. A checklist of 27 parameters, including the title, abstract, methods, results, discussion, and funding was taken into consideration to ensure the complete reporting of systematic reviews. PRISMA assures that authors prepare a transparent and complete reporting of systematic reviews and meta-analyses [27]. The PRISMA statement starts with identifying possible studies to include in our further revision after searching in several databases. We searched papers in the Scopus, Web of Science, and PubMed databases during June 2022. After testing several keywords, the search strategy used the term “patient satisfaction” to extend the number of results. Reference lists from the collected articles were also searched for additional articles. Overall, one thousand six hundred fifty-three studies composed the list of our first search.

Once we concluded our search, we removed the duplicates (241), and the remaining documents (1412) were analyzed under the inclusion and exclusion criteria. Inclusion criteria included: articles from peer-review journals; written in English; published from January 2000 to December 2021; assessed which factors affect patient satisfaction (or a proxy of it); evaluated overall patient satisfaction with healthcare; quantitative studies; reviews; and international studies to provide a more comprehensive analysis. We also excluded reports, books or book chapters, conference proceedings, dissertations, theses, expert opinion, commentaries, editorials, and letters. We excluded 1197 studies from the list after removing duplicates because they failed all inclusion criteria or met at least one exclusion criterion.

We conducted a full-text analysis to assess the eligibility of the remaining 215 papers. Disease-centered studies that did not evaluate the general aspects of patient satisfaction were excluded. We also discarded papers with unclear data collection methods, papers with unclear results, and qualitative papers. We rejected a total of 58 papers in this step.

**Figure 1:**
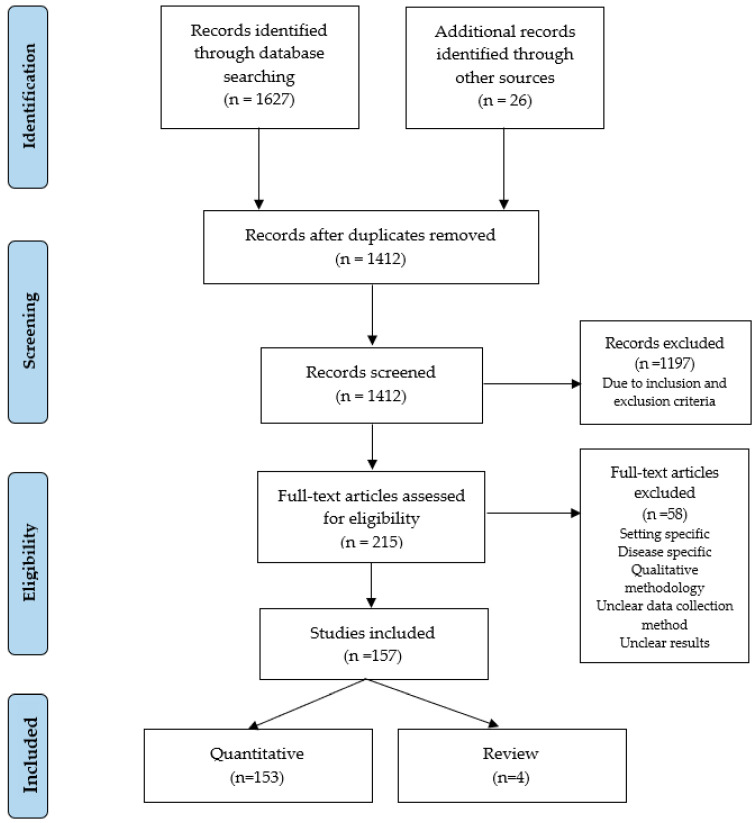
PRISMA statement.

Figure 1 outlines the PRISMA diagram detailing the study selection process. Following such a statement, 157 studies met the inclusion criteria. Four of them were systematic reviews, and the remaining 153 apply quantitative methods for patient satisfaction analysis.

## 3. Past Research on Patient Satisfaction: What Did the Systematic Reviews Tell Us about It?

The first systematic review [16] included a study of twenty-four articles published between 1978 and 2006. Through the analysis of these articles, health care output, access, caring, communication, and tangibles were the dimensions determining patient satisfaction. The patient’s socio-demographic characteristics that affected satisfaction were age, education, health status, race, marital status, and social class. SERVQUAL (service quality) was the most preferred instrument in satisfaction studies. In their review, the authors developed a conceptual model, claiming that healthcare quality and determinants influence patient satisfaction, influencing patient loyalty.

The second systematic review [17] included thirty-seven international articles from 2002 to 2013. Patient-professional interactions, the physical environment, and internal management processes were the most influential satisfaction constructs, except for specific services, such as home care, psychiatric or pediatric services. Almeida et al. disregarded patient’s socio-demographic characteristics from their review. In this type of service, phone contact and provided information were also considered powerful satisfaction constructs. The primary methodology used in the collected articles was factor analysis (exploratory factorial analysis (EFA) and confirmatory factor analysis (CFA)).

The third systematic review [14] comprised one hundred and nine international articles from 1980 to 2014. This study identified nine determinants of satisfaction: technical skills, interpersonal care, physical environment, accessibility, availability, finances, organizational characteristics, continuity of care, and care outcome. Technical skills constitute a cluster of medical care, nursing care, friendliness, concern, empathy, kindness, courtesy, and respect. The physical environment results from the atmosphere, room comfort, bedding, cleanliness, temperature, lighting, food, comfort, equipment, facilities, and parking. Accessibility is composed of location, waiting time, the admission process, the discharge process, and the effort to get an appointment. Availability represents the number of doctors, nurses, facilities, and equipment. Meanwhile, finances include the flexibility of payments, the status of insurance, and insurance coverage. Organization characteristics include reputation, image, administrative processes, doctors’ and nurses’ satisfaction level, and doctors’ socio-demographic characteristics. The authors found thirteen patient socio-demographic characteristics in their review: patient age, gender, education, socio-economic status, marital status, race, religion, geographic characteristics, visit regularity, length of stay, health status, personality, and expectations. The relationship between patient satisfaction and these socio-demographic characteristics was contradictory across the articles; thus, the authors achieved no conclusions. The most used methodology from the articles’ collection was not mentioned in the review.

Finally, the fourth and most recent systematic review [28] includes only thirty-eight international articles from 2000 to 2017. Provider’s attitudes, technical competence, accessibility, and efficacy were found to be the most influential attributes in this study. Provider’s attitudes comprise courtesy, friendliness, kindness, approachability, respect, responsiveness, attention, and concern. Accessibility also consists of a cluster of attributes, including the location, environment, equipment availability, appointment arrangement, and access equitability. Expectations, patient’s socio-demographic characteristics, and market competition were considered as antecedents to satisfaction while influencing it. There is no mention of the methods most used by the articles collected.

These four systematic reviews analyzed multiple articles to find the determinants of patient satisfaction. The gathered results and conclusions show coherence between the reviews. However, we can identify some limitations. The sample size of some reviews is somehow insufficient to achieve reliable conclusions. The methodology present in the articles collected should be studied more deeply to determine the different methodologies used in patient satisfaction studies and their advantages and disadvantages.

## 4. Results and Discussion

### 4.1. A Summary of Quantitative Papers Passing the PRISMA Sieve

After searching for quantitative papers within the patient satisfaction field, we constructed a table containing the most relevant data retrieved from them: author names, published year, country of study, sample size, satisfaction dimensions and drivers (if applicable), methodology, dependent variable, main factors affecting patient satisfaction, number of citations, and publisher. We considered a dependent variable field because, besides overall patient satisfaction, some articles studied proxies of satisfaction, such as willingness to recommend the hospital or willingness to return. Table 1 contains an excerpt of the articles with more than 100 citations (a total of 19 studies).

Table 2 and Table 3 present statistical measures applied to the data collected. The sample size, the first object of analysis, shows a significant coefficient of variance due to the values’ dispersion, as shown through the minimum and maximum rows in both analyses. In Table 2, the dispersion is more significant, with a higher coefficient of variation. The study with the most significant sample size in the first analysis is McFarland et al. [45], which analyzed 3907 private hospitals. For the second analysis, Aiken et al. [12] has the largest sample size.

Regarding the methodology used, most studies applied only one method. However, some studies used two methods in a complementary way. The number of criteria used to assess patient satisfaction has a low variance. Researchers give more importance to criteria than to explanatory variables through the values present in these tables. The number of criteria has a higher mean, median, and mode, meaning that researchers tend to disregard the vital aspect of satisfaction drivers. The main difference between both analyses is the minimum and the maximum number of criteria applied in the studies. Table 2 shows a minimum of zero and a maximum of 26, meaning that studies only assessed the importance of explanatory variables. However, in Table 3, the minimum is one, showing that studies with higher citations do not solely evaluate explanatory variables. The maximum number of criteria is also distinct in both tables, with a decrease of ten units in Table 3. Explanatory variables have a 100% coefficient of variance, with equal mean and standard deviation on both analyses. The number of critical factors has low variance, and the minimum is equal to one because each study seeks to fix the determinants of patient satisfaction. On the one hand, in Table 2, the number of citations presents a more dispersed pattern with a higher difference from the minimum to the maximum value. Over 20 years, it is reasonable to collect articles with an exact number of citations. On the other hand, Table 3, showing only articles with more than 100 citations, presents a more cohesive dataset.

### 4.2. Statistical Analysis over the Utilization and Importance of Satisfaction Criteria and Explanatory Variables

Factors related to satisfaction can be either criteria or explanatory variables. The assessment of hospital service quality can be a complicated task that includes numerous criteria, qualitative and dubious factors that are difficult to assess [46].

From the collected papers, we analyzed each factor’s utilization related to patient satisfaction. We also verified the importance rate of each factor. The percentage of utilization is the ratio between the number of studies using it is and the total number of evaluated studies, while the importance rate of a factor measures the relative number of papers concluding that this factor is critical for patient satisfaction.

In general, studies about patient satisfaction try to unveil factors associated with his/her overall satisfaction with one or more services (96% of the collected studies) or willingness to recommend the hospital/clinic (9%) instead. A smaller percentage of studies (7%) included both dependent variables [12,40,47,48,49,50,51,52]. There is one dependent variable (typically the overall satisfaction) explained by a series of criteria and other external factors. However, one can also use other dependent dimensions as proxies for such overall satisfaction. Examples include the willingness to return [36,48,53], medical services satisfaction, accommodations services satisfaction, nursing services satisfaction [54], satisfaction with the quality of medical information [55], and healthcare quality [56]. Compared with the overall satisfaction and the willingness to recommend hospitals/clinics, other studies are scarce within the sample of papers passing the PRISMA sieve. Therefore, we conducted three different analyses because of the different dependent variables used in each article, i.e., global analysis regardless of the dependent variable used, overall patient satisfaction, and willingness to recommend a hospital/clinic.

To provide a more unambiguous graphic representation of the analysis, we grouped some patient satisfaction related factors related to each other into a single factor. These are some examples: (i) concern (from the doctor, the nurse, or other staff, either clinical or not); (ii) clinical staff social characteristics (assurance, attention, attitudes, kindness, skills, and specialty); (iii) hospital characteristics (image, location, quality, size, and type); and (iv) patient’s social characteristics (autonomy, dignity, emotional support, income, life expectancy, marital status, nationality, occupation, race, residence, satisfaction with life, and stress level).

#### 4.2.1. Global Analysis about the Most Frequently Used Satisfaction Criteria and Explanatory Variables

**Figure 2:**
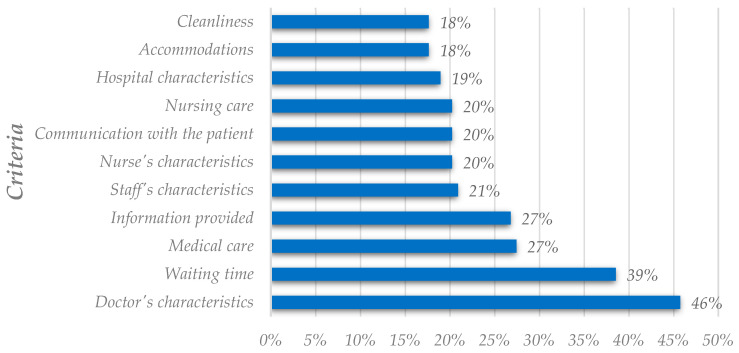
Analysis of the most utilized criteria in the literature (global analysis).

**Figure 3:**
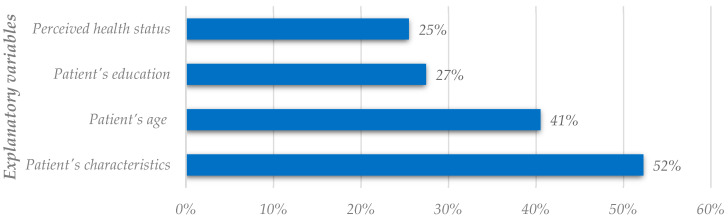
Analysis of the most utilized explanatory variables in the literature (global analysis).

We started by analyzing all factors related to patient satisfaction, clustering in terms of satisfaction criteria and explanatory variables, regardless of the dependent variable used by researchers. As their name indicates, explanatory variables are useful to find out potential drivers or determinants for satisfaction. They are nondiscretionary for hospital/clinic managers but may play a prominent role in infrastructure management. We divided the fifteen most utilized factors into criteria and explanatory variables; Figure 2 and Figure 3 represent them, respectively. These factors are the ones that most researchers use to study patient satisfaction and may not correspond to the most important and influential factors of patient satisfaction.

Of the fifteen most used factors, eleven are criteria, and four are explanatory variables. The doctor’s characteristics, waiting time, medical care, and information provided have the highest utilization rates of utilization within the criteria. Patient’s social characteristics, patient’s age, patient’s education, and perceived health status also have the highest utilization rate, but are within the explanatory variables. However, this analysis is not directly related to the importance rate analysis; thus, the most utilized factors might not be the most important and influential.

**Figure 4:**
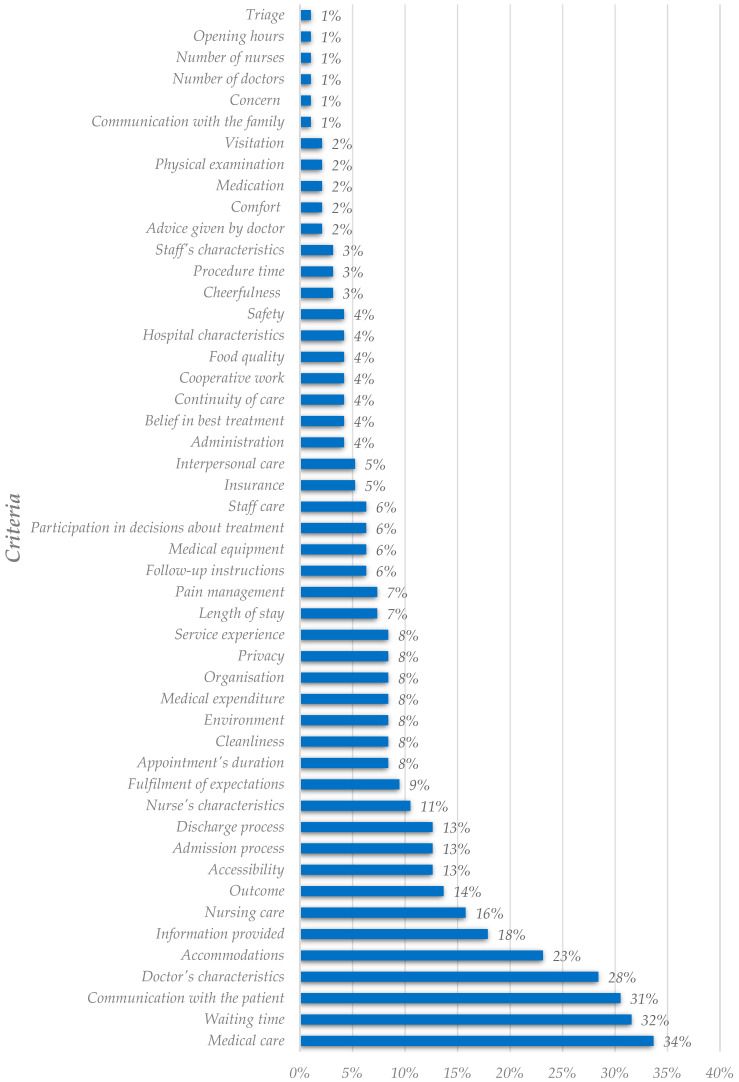
Analysis of criteria deemed as the most critical in the literature (global analysis).

**Figure 5:**
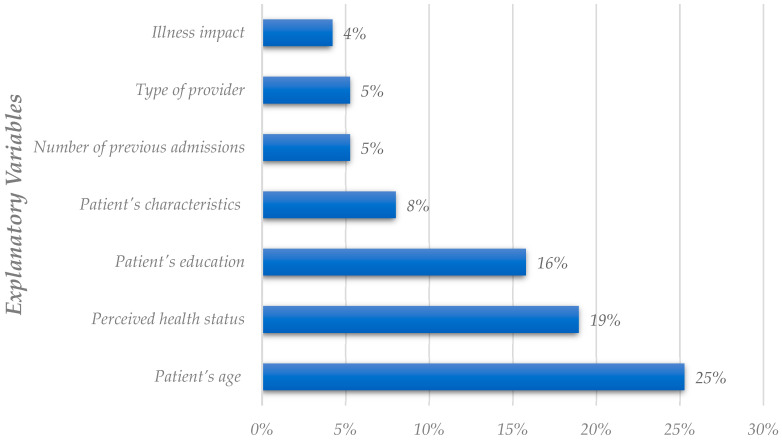
Analysis of explanatory variables deemed as the most critical in the literature (global analysis).

Figure 4 ranks the criteria deemed as the most important to evaluate satisfaction. In contrast, Figure 5 presents the most critical explanatory nondiscretionary dimensions. This first analysis, the most complete one, resulted in fifty-six factors, divided into forty-seven criteria and nine explanatory variables. From Figure 4, it is possible to conclude that the three criteria most important in collecting articles are medical care, waiting time, and communication with the patient. Despite not being in the top three, criteria related to the doctor’s social skills exhibit a high importance rate and should be noticed. It is interesting to note that researchers conclude that staff’s social skills, such as communication, are more critical than others, such as like food quality and comfort. Also, the criteria associated with the technical skills of staff appear to be less critical. This seems to be in line with some authors who claim that patients are usually unable to judge health professionals in those terms [2,3,4,5].

Additionally, waiting time is one of the most critical criteria to study patient satisfaction. For instance, Ferreira et al. [25] classified this criterion as a critical must-have requirement. It means that patients take it for granted and neither get satisfied nor dissatisfied if the waiting time is null. However, their dissatisfaction increases intensely when waiting time becomes more substantial. The authors also verified that waiting time was the most crucial criterion for patients in medical appointment services.

Figure 5 shows that the patient’s age, the perceived health status, and the patient’s education are the variables that studies tend to consider as the most influential to patient satisfaction. Previous studies that say that age, education, and self-reported health status have an evident and significant influence on the satisfaction outcomes were confirmed [38]. Older patients or those with better self-perceived health status are typically more satisfied, while highly educated people are less satisfied with the healthcare services provided [34,42].

Differences arise after comparing the results from the utilization analysis and the importance rate analysis. Figure 2 and Figure 4, both portraying criteria, reflect differences in the ranking positions. The doctor’s characteristics, the most utilized criterion, was placed fourth on the importance-related raking. Communication with the patient also occupies different positions in the analysis. Figure 2 shows this criterion in the seventh position, while in Figure 4, it is the third criterion with the highest importance rate.

Regarding the explanatory variables, Figure 3 and Figure 5 also display disparities. The patient’s social characteristics are the most used cluster of explanatory variables, but it occupies the fourth position on the importance rate analysis. The patient’s age has the second-highest utilization rate and the highest importance rate. The patient’s education occupies the third position in both analyses. Lastly, perceived health status is ranked fourth in Figure 3, but secondly in Figure 5.

#### 4.2.2. Overall Patient Satisfaction as the Dependent Variable

As in the previous case, we divided our analysis into criteria and explanatory variables. Identical to the global analysis, the fifteen factors deemed the most used in literature are eleven criteria and four explanatory variables. The results of this analysis are equal to the global analysis’ results, with a slight change in the percentage level. The doctor’s characteristics, waiting time, medical care, and information provided remain the most used criteria with a percentage of 43%, 38%, 28%, and 27% each. The four explanatory variables continue the same, with the patient’s characteristics, patient’s age, patient’s education, and perceived health status as the most utilized. The percentages of these variables suffered minor modifications.

Regarding the importance analysis, fifty-six factors are assessed, similar to the first analysis, with forty-seven criteria and nine explanatory variables. One can only observe a few differences between this and the previous analyses. It is mostly motivated by the fact that researchers often tend to look at the overall satisfaction instead of other related concepts like willingness to return or recommending the healthcare provider. The three most important criteria are medical care, waiting time, and communication with the patient. These are the same as the first analysis, but with a modification of each criterion’s percentages. As mentioned above, the doctor’s social skills also have a similar percentage as the top three global analysis criteria. However, in this second analysis, the criterion “accommodations” has a higher utilization percentage. The patient’s age, perceived health status, and patient education are the most important explanatory variables. The variables are the same as in Figure 5 but have different percentages.

#### 4.2.3. The Willingness to Recommend the Healthcare Provider as the Dependent Variable

**Figure 6:**
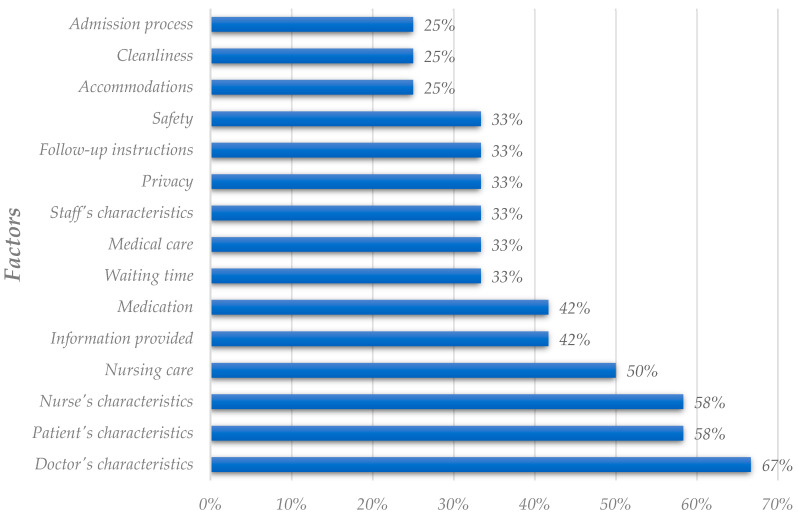
Analysis of the most utilized factors in the literature (willingness to recommend analysis).

The third and last analysis regards the dependent variable “willingness to recommend.” For the utilization analysis, we also present the fifteen most used factors. However, since one sole factor is an explanatory variable (patient’s characteristics), both criteria and explanatory variables are presented on the same chart (Figure 6).

The results of this utilization analysis differ from the two utilization analyses presented above. Despite doctor’s and patient’s characteristics being the factors with the highest utilization rates (consistent with the previous analysis), the remaining factors and percentages suffered alterations. Waiting time and medical care, the second and third criteria with the highest utilization rates on the previous analyses, are now positioned in seventh and eighth place, respectively. Nurses’ characteristics and nursing care are the criteria with the most significant rate increase, at more than 30%. The change in the dependent variable is responsible for the alterations of these results. Studies that consider the dependent variable “willingness to recommend” tend to adopt different factors, focusing on nursing care and professionals, compared with the studies analyzing the “overall patient satisfaction.”

**Figure 7:**
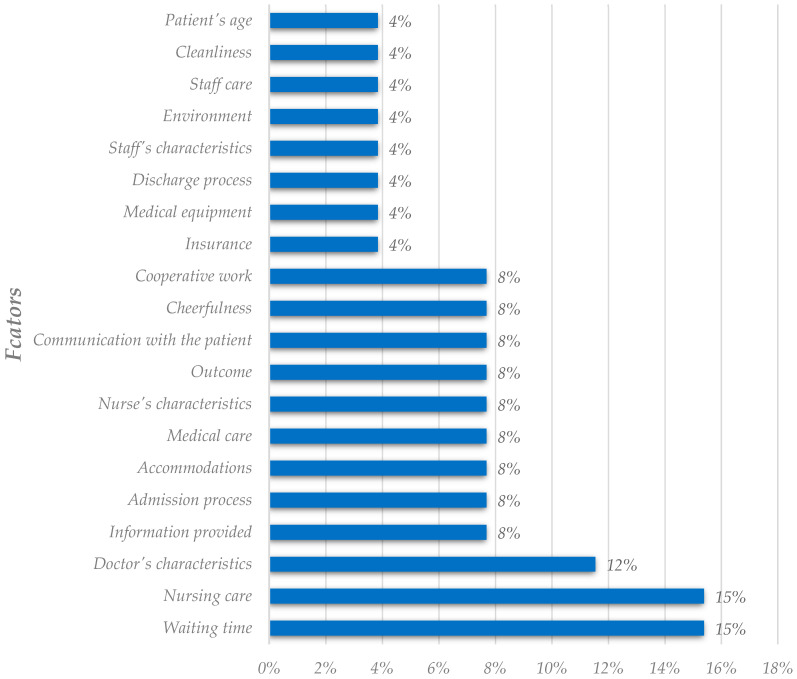
Analysis of factors deemed as the most critical in the literature (willingness to recommend analysis).

In the importance analysis, since there were only twenty factors assessed, including nineteen criteria, and only one explanatory variable, we show the results compiled in Figure 7. Regarding the explanatory variables, only the patient’s age resulted from our search. It is a consistent result compared to the previous ones, given that the patient’s age is the most relevant explanatory variable in all three analyses. As one can see, the three most important criteria are waiting time, nursing care, and the doctor’s social skills. Medical care, the most frequent criteria in the other two analyses, is not as relevant in this analysis. Nursing care is more relevant in this analysis than in the previous ones. It is in line with the results of the utilization analysis for this dependent variable. Nursing related factors are more frequent and deemed crucial when the dependent variable is “willingness to recommend.”

### 4.3. Discussion on the Most Critical Patient Satisfaction Criteria and Explanatory Variables

When comparing the results from the three analyses carried out, it is possible to reach conclusions about each factor’s importance. According to the literature, the criteria of medical care, communication with the patient, and waiting time are the three most critical. The doctor’s social skills, accommodations, nursing care, and information provided can also be considered relevant criteria. The explanatory variables of patient’s age, perceived health status, and patient’s education are the remaining ones.

Some past systematic reviews have revealed that interpersonal or social skills (such as medical/nursing care and attitudes), technical skills, infrastructure and amenities, accommodations, environment, accessibility, continuity of care, and the outcome are the satisfaction criteria present in the majority of studies related to satisfaction in healthcare [14,16,17,18]. In terms of explanatory variables, these reviews also point out the frequent use of variables such as the patient’s gender, age, education, and marital status. These dimensions should affect customers’ satisfaction ratings, helping to understand them, but should not be confused with criteria.

Despite the similarity of results between the previous reviews and the current one, some other factors seem to assume a high relevance for researchers. They include waiting time and the information provided, which interestingly are not present in the previous reviews. On the one hand, waiting time is a determinant of healthcare dissatisfaction, regardless of the inpatient stage. Waiting time is clearly an obstacle to access. Meanwhile, efficient hospitals usually have short waiting times [15]. The longer the waiting time, the more dissatisfied the customer is [57]. However, the converse is not necessarily true. If the waiting time is very short or even null, the customer may take it for granted because she/he needs the medical/nursing procedure and be neither satisfied nor dissatisfied. It means that waiting time is usually pointed out as a must-have requirement [25].

On the other hand, the criterion information provided may refer to any care process, since the patient enters the system until he/she leaves it. Communication or the capacity of providing useful information includes the treatment guidelines, rights, duties, means of complaint and suggestions, current health status, and the post-discharge process at home. For instance, inadequate post-discharge care and lack of patients’ preparedness are two potential determinants of readmissions for further care [58]. Readmissions within a specific (short) period after discharge may reveal a lack of care appropriateness, either in terms of the clinical staff’s technical skills or the information provided about care at home. Therefore, it is an excellent practice to sufficiently prepare the discharged patient (or someone responsible for her/him) for proper care at home. Missing or confusing information provided by the clinical staff contributes to a lack of preparedness and, by consequence, to customer dissatisfaction. We should remark that communication should be a social skill of any healthcare worker. The fact that this criterion does not appear in previous reviews is perhaps the result of merging some criteria related to it. However, we point out the need for high discrimination of criteria during a satisfaction survey.

### 4.4. Methods Employed in the Literature

**Figure 8:**
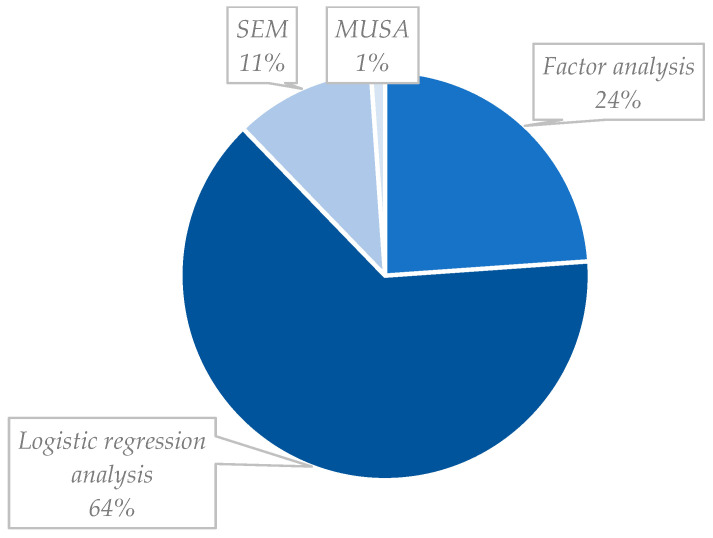
Analysis of methods utilized in the literature.

Figure 8 provides a chart comparing the different literature methods devoted to the patient’s satisfaction analysis. We identified four main methods: logistic regression analysis, factor analysis, structural equation modeling (SEM), and multiutility satisfaction analysis (MUSA). Nonetheless, other ancillary methods are suitable for satisfaction analysis when complementing those four.

We can observe that regression analysis is chosen by most researchers (64%). Factor analysis is in second place, with 24% utilization, followed by SEM (11%), and lastly MUSA (1%). From the 153 collected articles, 27 (18%) combined different methods in a complementary nature: factor analysis with regression analysis (16 of the 27 articles, or 59%), and factor analysis with SEM (11 of the 27 articles, or 41%), are the two combinations observed. The difference in the level of utilization of each method can be due to the difficulty of implementation. SEM and MUSA are more complex than the other two and thus harder to implement. Contrary to this, logistic regression and factor analysis are more straightforward to implement, becoming more attractive to the researcher. We provide a brief description of each method below to better understand the methodology used in the articles collected.

Factor analysis: A mathematical model explains the correlation between a broad set of variables in terms of a small number of underlying factors [59]. It uses procedures that summarize information included in a data matrix, replacing original variables with a small number of composite variables or factors [60].

Logistic regression analysis: This analysis is frequently employed to model the association between a response and potential explanatory variables. Every association is evaluated in terms of an odds ratio [61].

Structural Equation Modelling (SEM): This is a general modeling technique used to test the validity of theoretical models that define causal and hypothetical relations between variables [62,63]. Some researchers have combined SEM with other types of analyses for the study of satisfaction, although without an application to the case of healthcare, such as with Ciavolino et al. [64] and Sarnacchiaro et al. [65].

Multicriteria Satisfaction Analysis (MUSA): The basic principle of MUSA is the aggregation of individual judgments into a collective value function, assuming that customers’ global satisfaction depends on a set of criteria representing service characteristic dimensions [25,66]. MUSA has a generalization, called MUSA-INT [67], which accounts for positive and negative interactions among criteria.

### 4.5. Discussion on the Most Frequent Model/Method Used in Literature

In general, when analyzing the patient’s satisfaction, researchers tend to look at the relationship between overall satisfaction and partial satisfaction regarding each criterion. This implies the utilization of a multivariate model, which depends on regressions in most of the cases. We should point out a significant pitfall present in most of the empirical applications. Customers are typically asked to judge the healthcare provider in all possible dimensions using rating-like systems, which can be semantic expressions (e.g., very dissatisfied/…/delighted), symbols (e.g., 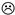/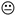/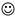), or numbers (e.g., 1/…/7). However, these numbers do not have the same meaning as numerals used in other real-life situations (as in economics and finance). They only carry an ordinal meaning (7 is better than 6, which is better than 5, and so on), but operations such as 1 + 2 or 2 × 3 are objectionable in mathematical terms. Therefore, some multivariate models are not suitable for dealing with this kind of data. This is the case with regard to SEM and factor analysis, which are still used by some researchers (35% of the collected papers). Indeed, they undertake mathematical operations that are not consistent with Stevens’ theory and data categorization [68]. We point out the existence of the so-called Cat-PCA method, which is a version of PCA (factor analysis) designed explicitly for dealing with the case of categorical data, such as the ones associated with satisfaction, as seen in Valle et al. [69] and Vuković et al. [70].

As seen in Figure 8, the most used method is logistic regression analysis due to its implementation simplicity and reliability of results. Logistic regression is a statistical method where one variable is explained or understood based on one or more variables. The variable being explained (typically the overall satisfaction or the willingness to return) is the dependent or response variable. The other variables used to explain or predict the response are the independent variables. Essential features of logistic regression include: (a) it provides a single regression coefficient estimate of covariates for each response category; (b) it follows stochastic ordering; (c) it is easy and straightforward to apply; (d) it needs a few parameters to estimate; and (e) the odds are proportional across the response variable are [71,72]. The outcome variable in an ordinal logistic regression model can have more than two levels. An estimation of the probability of being at or beneath an outcome level, depending on the explanatory variable, is executed in this analysis [73]. Limitations are, however, also a part of this model, since: (a) large samples are required since the coefficients are estimated by maximum likelihood estimate; (b) proportional odds assumption should be satisfied, meaning that the odds ratio is constant across the cut-off point for each of the covariates in the model. If this assumption is not truthful, the estimate of the parameters obtained is not valid [71].

Another alternative for categorical variables is MUSA [66]. This model can be applied to the satisfaction-related data [74,75,76] because it estimates (through optimization) the value functions associated with each criterion and value scales are no longer categorical. Unlikely logistic regressions, MUSA does not rely on the proportional odds assumption that rarely is satisfied in practice. In truth, MUSA is a model for satisfaction analysis, which is nothing but a robust ordinal regression. For that reason, we may argue that MUSA is the non-parametric version of logistic regression and, therefore, makes fewer assumptions than the latter. In other words, while some validity conditions must be met so that the result of parametric models is reliable, non-parametric models can be applied regardless of these conditions. However, the power of the parametric model is traditionally superior to the power of its non-parametric counterpart. Furthermore, MUSA is challenging to implement, limiting its use by more researchers. We should point out that it is good practice to apply several alternative models (namely MUSA and the logistic regression) and to test for the robustness of outcomes.

Other approaches have also been proposed in the literature to study satisfaction. One is the so-called Benefit of Doubt model [77], which is a particular case of the well-known Data Envelopment Analysis (DEA). Bulut [78] used the Benefit of Doubt to construct a composite index based on citizens’ emotions and senses. Such an alternative was not applied to the healthcare sector at that point in time. However, Löthgren and Tambour [79], Bayraktar et al. [80], Gok and Sezen [81], Mitropoulos et al. [82], and Mohanty and Kumar [83], to name a few, have included satisfaction data in their DEA exercise. The same critique made to the use of ordinal data in SEM and PCA applies to the case of the Benefit of Doubt (and DEA), as it is nothing but a linear programming model. The Benefit of Doubt is commonly used for benchmarking purposes. If one wishes to benchmark decision-making units based on satisfaction, one can couple MUSA and Doubt’s benefit. Indeed, recalling Grigoroudis and Siskos [66] and Ferreira et al. [25], one can establish a satisfaction index associated with each criterion and decision-making unit. Such an index is a function of the utilities per satisfaction level. Therefore, these indexes can replace the indicators traditionally used in the Benefit of Doubt model. Another alternative is the DEA-based *maxmin* model of Li et al. [84], Dong et al. [85], and Wu et al. [86] that allows the maximization of each decision-making unit’s satisfaction.

The principal component logistic regression is another alternative recently proposed. According to Lucadamo et al. [87], Labovitz [88] and O’Brien [89], “*proved that if the number of categories is sufficiently large (e.g., six or seven points), one can apply the product-moment correlations on ordinal variables with negligible bias*.” However, such conclusions resulted from controlled simulation procedures, which might hardly apply to the real world. Although the logistic regression has its own merits regarding the analysis of satisfaction determinants, it also has limitations, as discussed above. Unless “*successive categories of the ordinal variables are equally spaced*” (an extreme assumption), then the merging of PCA and logistic regression is not likely to produce reliable results.

The multiobjective interval programming model proposed by Marcenaro-Gutierrez et al. [90] and Henriques et al. [91,92] which was applied to explore the trade-offs among different aspects of job satisfaction is an interesting one. In short, the model optimizes some coefficients related to those trade-offs by merging interval programming and econometric techniques. It should be explored in the future and applied to the healthcare sector.

### 4.6. Is There Any Association between the Adopted Method and the Criteria Deemed More Critical for Satisfaction Analysis?

To corroborate or invalidate the hypothesis of an association between adopted methods and critical factors, an analysis was performed where the critical factors mentioned per study were clustered in terms of the method adopted. As demonstrated above, the main critical factors are waiting time, medical care, communication with the patient, information provided, and patient’s age. Logically, these factors are the most prominent in each method, given their high importance rate.

From the logistic regression analysis, waiting time, patient’s age, communication with the patient, doctor’s characteristics, and medical care are the factors with the most notable presence. In factor analysis, doctor’s characteristics, medical care, waiting time, the information provided, and accommodations are the most relevant factors. Using SEM, accommodations and doctor’s characteristics are the more noticeable factors. Finally, with MUSA, accommodations, waiting time, doctor’s characteristics, and admission process are the most distinguishing factors. However, due to the reduced number of studies applying this latter method, it was not possible to assess any pattern of association between this method and the critical factors.

Assessing the analysis results, it is possible to conclude that most factors with a consistent presence within the different methods are critical factors. Thus, we found no association pattern between the most critical factors and the researcher’s method.

### 4.7. Is There Any Association between the Country and the Critical Factors?

To examine a possible relationship between the country of study and the critical factors, an analysis was performed on the five countries with the highest number of studies. This restriction was applied because of the reduced number of studies per country and the inability to reach conclusions with a reduced number of studies. The USA, the country with a higher number of studies, revealed that patients’ most critical factors are doctor’s characteristics, patient characteristics, waiting time, and patient’s age. In Germany, doctor’s characteristics, organization, outcome, and medical care are the most critical factors. Chinese patients consider medical expenditure and doctor’s characteristics to be the two most important factors. In Portugal, accommodations, waiting time, accessibility, and medical care are deemed the most critical factors. Finally, in Turkey, doctors’ and nurses’ characteristics and waiting time are the most critical factors for the patients.

It is possible that factors diverge from country to country, giving insight into patients’ preferences from different parts of the world [93]. The doctor’s social skills are the most important for most countries, followed by waiting time and medical care.

## 5. Bibliometric Analysis

Bibliometric analysis is a separate analysis that one can apply to evaluate research by analyzing bibliographic data and describing publication patterns within a determined field. Methods such as co-citation and bibliographic coupling, which are discussed below, can be considered relational techniques to explore research structure, indicating patterns of authors or affiliations and prominent topics or methods [94,95]. The number of articles included in this bibliometric analysis differs from the number included in the statistical analysis. For the collection of articles, the databases Scopus, Web of Science, and PubMed, as already mentioned, were all explored. However, when performing a bibliometric analysis, it was not possible to compile the citation files of different databases. Thus, to achieve and extract the maximum information possible, we choose the Scopus citation file as the one with the highest potential to perform the bibliometric analysis in the Bibliometrix R package software. The citation files have different sizes, the Scopus database being the one with the highest number of articles included, 140. Web of Science included 118, and PubMed only included eight articles. It is important to note that the same article can be present in more than one database.

**Figure 9:**
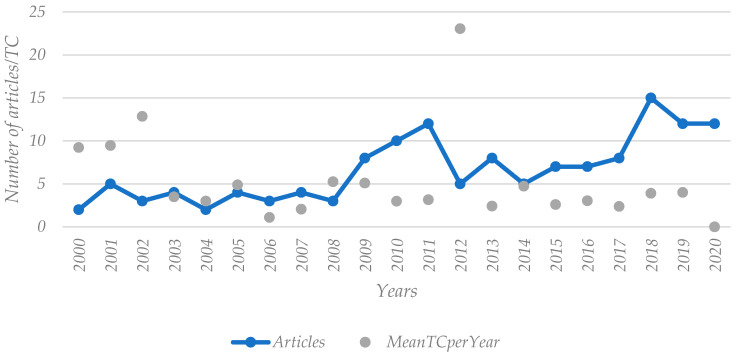
Number of articles and mean of total citations (TC) per year.

Figure 9 presents the growth rate of the number of published articles and mean total citations (TC) in the collection. It can be observed that these two variables are not aligned with each other, meaning that when one grows, the other does not necessarily grow as well. In the years 2009, 2010, and 2011, the number of published articles peaked, with a percentage of 6%, 7%, and 9%, respectively. The number of publications then decreased and peaked in 2018, 2019, and 2020. The mean of TC reached peaks in 2000, 2011, 2002, and 2012, and thus was not directly related to the number of publications. Neither of these variables follows a linear path, since the inflation occurs in what seems to be random intervals of time.

In this collection of 140 articles, 100 journals served as sources. The ten journals displayed in Table 4 are the ones with the highest number of publications (in this collection) and alone represent 42% of the articles. Most of the journals are related to health, as would be expected, but patient satisfaction is multidisciplinary. Despite many articles related to health, business, social sciences, and biochemistry, there are examples of subject areas in which this topic is included. When comparing the number of TC for each journal with the articles in this collection, *Social Science & Medicine* is the journal where the highest cited articles are published.

In total, there is a reach of 57 countries in this review. However, only the ten countries with more publications are presented in Table 5. It is noticeable that most of the articles collected are from the USA. Germany, China, and Portugal also have a particular emphasis, being the countries with the highest number of publications. It is important to note that many publications have affiliations with more than one country. Among the ten most impactful institutions, four are from the USA, and the remaining are also from countries mentioned in Table 5.

### 5.1. Co-Citation Analysis

A co-citation analysis measures the frequency of two publications cited together, indicating the affinity and proximity between them [96], and can also be applied to authors and sources. Two documents are co-cited when a third document cites them; having more documents that cite the same two documents translates into a more robust association [95]. This analysis says that two co-cited papers have a similar theme [94], thus identifying the most influential authors and their interrelationships inside a determined theme [97]. The co-cited papers are grouped into different clusters, considering the research area’s knowledge base and the similarity of themes [98]. The results of the top fifteen publications are presented in Table 6.

Table 6 provides a citation analysis to identify the most influential articles on the subject of patient satisfaction. In addition to the total number of citations, the number of average citations per year is also included in this analysis, given that it provides an unbiased look at the impact of each article without prioritizing the year of publication [99,100]. When analyzing Table 6, it is possible to conclude that the article with the highest number of citations is the most impactful and influential in the collection. The same happens for the following four articles. An example of an article with a ratio of TC/Year that is relatively high for the number of TC is Bjertnaes et al. [43], ranked on the second to last table position.

#### 5.1.1. Documents’ Co-Citation Analysis Results

**Figure 10:**
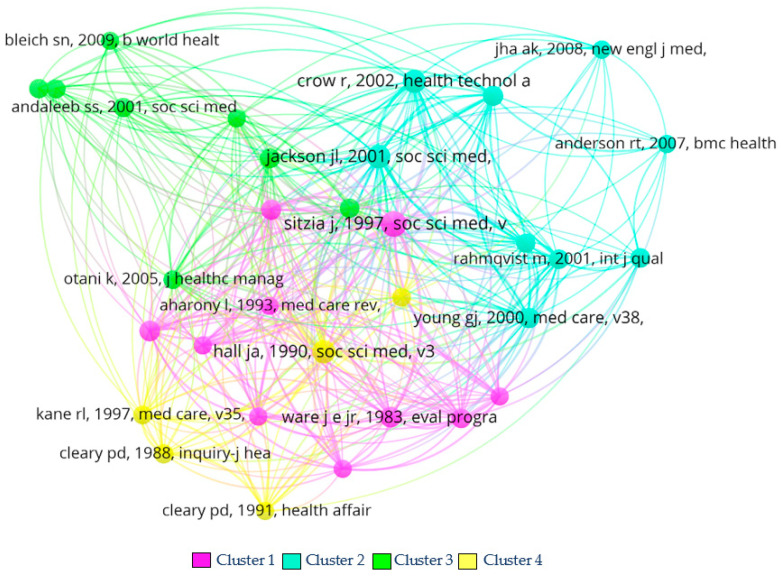
Documents’ co-citation analysis network using VOSviewer software. Source: authors’ own construction.

Figure 10 shows the network of the co-citation analysis (the graphs are constructed through VOSviewer software). Circles represent the items (documents, authors, sources, or keywords). The higher the number of citations or occurrences, the larger the size of the circle. Not all items are displayed; otherwise, they might overlap. The path length estimates the distance between items; the closer two items are, the stronger their relatedness. The connections between the items (lines) are called links. Link strength is a number associated with the link itself, which shows how secure the connection is between the items assessed. For instance, in the co-authorship links, the higher the link strength, the higher the number of publications the two authors developed [101,102].

Through association strength and considering publications with a minimum of seven citations, 32 articles within four clusters were found. It is important to note that the citations considered are local citations.

**Cluster 1** comprises ten articles and is the second most cited cluster, with 102 citations and the most important regarding link strength (566). The studies included in this cluster are from the USA and are mainly reviews or academic settings. The main article within this cluster is Sitzia and Wood [103] with 24 citations and a 133-link strength. This cluster has the oldest set of collected articles, all ranging from the 1980s to the 1990s.

**Cluster 2** is a collection of nine articles, with 103 citations and 485 link strength. Overall, it is the cluster with the highest number of citations but the second in link strength. These studies are all from the USA and create more profound research since they explore what factors influence patient satisfaction by developing patient surveys. The articles with the most impact in this cluster are Jackson et al. [11] with 18 citations and 94 link strength, and Jaipul and Rosenthal [104], with ten citations and 62 link strength. The majority of articles presented in this cluster are from dates after the 2000s.

**Cluster 3**, in its turn, is composed of eight articles, with a total of 57 citations and 218-link strength. This cluster has the lowest number of citations and is the second to last regarding link strength. The articles focus on finding the factors that influence patient satisfaction, but are not as in-depth as the second cluster articles. Some articles with an academic setting are also present. The most important articles in this cluster are Andaleeb [32] with nine citations and 31 link strength; Parasuraman et al. [105], with nine citations and 23 link strength; and Otani et al. [106], with eight citations and 44 link strength.

**Cluster 4** has five articles, with a total of 249 citations and 50 link strength. Regarding citations, this cluster is ranked in third place. It is the least important in terms of link strength. The articles from this cluster are all from the 1980s and 1990s, similar to the first cluster. Hall et al. [107], with 18 citations and 104 link strength, Cleary and McNeil [108] with nine citations and 39 link strength, and Kane et al. [109] with eight citations and 37 link strength, are the articles with the most impact in this cluster.

#### 5.1.2. Authors’ Co-Citation Analysis Results

**Figure 11:**
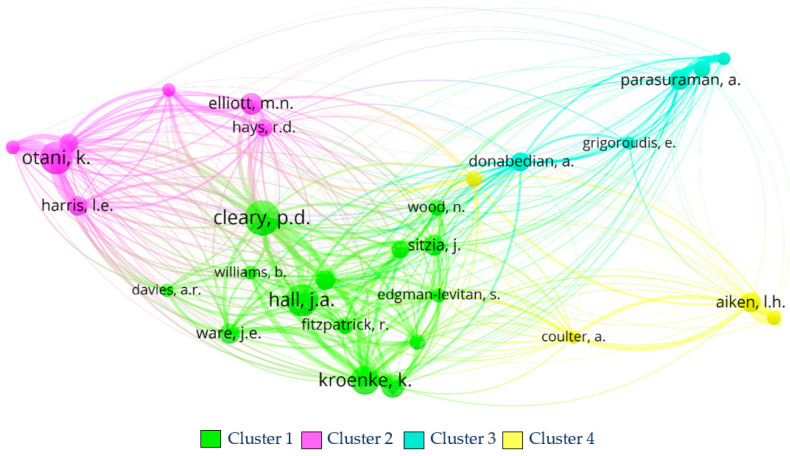
Authors’ co-citation analysis network using VOSviewer software. Source: authors’ own construction.

There are 8691 authors; thus, a restriction of a minimum of 20 citations per author was applied, and 30 authors were found divided into four clusters. Figure 11 shows the network of the author’s co-citation when the method of association strength is applied. The circles’ size represents the number of times the author is cited in the collection of 140 articles.

**Cluster 1** includes fourteen authors and is the most crucial cluster, with the highest number of citations (449) and link strength (4775). The most relevant authors are Cleary (54 citations and 581 link strength), Hall (50 citations and 466 link strength), and Kroenke (45 citations and 535 link strength). These authors correspond to cluster 4 of Figure 10, highlighting two of the above mentioned as the authors of two of the most cited documents of cluster 4 on the documents’ co-citation analysis.

**Cluster 2** includes seven authors and is placed second on ranking the number of citations (213) and link strength (2147). Otani is the most relevant author in this cluster (50 citations and 485 link strength). This author is also the one with the most contributions to the collection, with a total of six articles, followed by Elliott (34 citations and 243 link strength) and Kurz (29 citations and 337 link strength), who collaborates with Otani on four of the six articles present in the database. Some of these authors have articles present on cluster 3 of the co-citation analysis.

**Cluster 3** has five authors and placed third with regard to citation’ ranking (125) and link strength (969). Parasuraman (31 citations and 228 link strength), Donabedian (29 citations and 275 link strength), and Zeithaml (25 citations and 205 link strength) are the most influential authors in this cluster. The authors of this cluster are dispersed through clusters 3 and 4 of the documents’ co-citation analysis.

**Cluster 4** is composed of four articles and is the least relevant cluster in citations (99) and link strength (823). The authors in this cluster are Aiken (31 citations and 236 link strength), Orav (25 citations and 185 link strength), Sloane (22 citations and 199 link strength), and Coulter (21 citations and 203 link strength). These authors are not present in Figure 11 due to the low number of local citations of their articles.

#### 5.1.3. Sources’ Co-Citation Analysis Results

**Figure 12:**
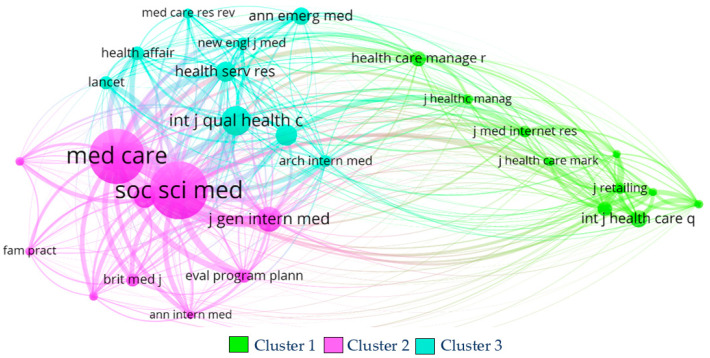
Sources’ co-citation analysis network using VOSviewer software. Source: authors’ own construction.

In Figure 12, sources (journals) where the articles were published are assessed to find the frequency in which two sources are co-cited. It translates into the similarity between the scope of focus of the sources. Each circle represents a journal, and the size of the circle is proportional to the number of citations. Sources in the same cluster or that are connected have similarities.

From the 2278 sources present in the sample’s references, only sources with more than 20 citations were considered, making up a total of 29 sources. Through the method of normalization strength, three clusters were found. It is important to note that the sources in this analysis are the sources of documents cited by the collection of 140 articles and not the sources from the 140 articles in the collection.

**Cluster 1** is composed of ten items and is the least important in citations (303) and link strength (4879). Despite being one of the most significant clusters, the sources included are not the most renowned. *The International Journal of Health Care Quality Assurance* (47 citations and 614 link strength), *The Healthcare Manager* (41 citations, and 496 link strength), and *The Journal of Marketing* (40 citations and 767 link strength) are the most relevant journals in this cluster. Cluster 1 is the only cluster of the analysis that included sources not only related to the medical field, such as the *Journal of Marketing*, *Journal of Retailing, Journal of Services Marketing, Journal of the Academy of Marketing Science*, and *Managing Service Quality*.

**Cluster 2** has ten items and is the most important regarding citations (660) and link strength (9719), as can be seen by the differential size of the circles in Figure 12. *Social Science & Medicine* (189 citations, and 2632 link strength), *Medical Care* (179 citations and 2450 link strength), and *The Journal of General Internal Medicine* (73 citations and 1217 link strength) are the most important sources regarding citations’ link strength in this cluster. Special attention goes to *The British Medical Journal, Annals of Internal Medicine*, and *The Journal of the American Medical Association*, which are the sources with a significant impact on the field. It is noticeable that the second cluster is the most important and impactful of the three.

**Cluster 3** included nine sources and is in second place in citations (400) and link strength (4890). *The International Journal for Quality in Health Care* (86 citations and 995 link strength), *BMC Health Services Research* (59 citations and 683 link strength), and *Health Services Research* (56 citations and 756 link strength) are the sources with the most citations and link strength in this cluster. It is necessary to give a particular highlight to other sources integrated into this cluster, such as *The New England Journal of Medicine* and *The Lancet*, two highly influential journals in medicine.

### 5.2. Discussion on the Bibliometric Analysis Results

After conducting a bibliometric study with co-citation and bibliographic coupling analysis, it is possible to assess the changes and constants on patients’ satisfaction studies throughout the years and the most influential articles, authors, and journals in the field. The research topic is constant throughout the years, discovering the factors that influence patient satisfaction, those being explanatory variables or criteria that remain in focus. Being that this a worldwide study, articles from multiple countries and institutions were analyzed. However, the USA is the country with the highest number of published articles in this field and the largest number of institutions that executed the research. From a collection of 140 articles, 39% are from the USA, followed by China and Germany, with 8% each. The discrepancy between the number of articles in the USA and the remaining countries might be a result of their health system. Since most facilities are for-profit organizations, it is imperative to keep the customers (patients) satisfied, secure their loyalty, and, thus, ensure the organization’s success. Profitable loyalty and satisfaction need to be taken to a higher level where differentiation and competitive advantage are met [110].

As mentioned above, with bibliometric methods, it is possible to assess which articles, authors, and journals are the most important in the subject area. Combining the results from both co-citations and bibliographic coupling analysis ensures that a large number of items are evaluated and that definitive conclusions can be achieved.

For the co-citations and bibliographic coupling analysis of articles, the top five documents, considering citations and link strength are: *Patient satisfaction: A review of issues and concepts* by Sitzia and Wood [103], *Predictors of patient Satisfaction* by Jackson et al. (2001), *Patient sociodemographic characteristics as predictors of satisfaction with medical care: A meta-analysis* by Hall and Dornan [111], *Patients’ experiences and satisfaction with health care: results of a questionnaire study of specific aspects of care* by Jenkinson et al. (2002), and *Service quality perception and patient satisfaction: A study of hospitals in a developing country*, by Andaleeb [32]. From the decades of 1990 and 2000, these documents are the most influential with regard to the topic of patient satisfaction. It is important to note that from Table 6, the document *Patient safety, satisfaction, and quality of hospital care: Cross-sectional surveys of nurses and patients in 12 countries in Europe and the United States* from Aiken et al. [12], is the article with the most citations, as well as the most influential article throughout the years. However, it was not included in the co-citation nor the bibliographic coupling analysis because it had no connection with the other articles, and thus zero link strength.

The authors’ co-citation and bibliographic coupling analysis revealed that the five most influential authors regarding citations and link strength are Cleary, Hall, Otani, Sjetne, and Aiken. Even though these authors do not have articles presented above that are mentioned as the most influential, these authors have written multiple articles on patient satisfaction and are thus essential and influential in the field.

The sources’ co-citation and bibliographic coupling analysis show that in terms of citations and link strength, *Social Science & Medicine* and *Medical Care* are the two most prestigious journals. *The International Journal for Quality in Health Care, The Journal of General Internal Medicine*, and *The Journal of the American Medical Association* are also influential journals in patient satisfaction.

## 6. Concluding Remarks and Future Directions for Research

This study reviewed studies published between 2000 and 2021 in three databases regarding patient satisfaction determinants. Firstly, a statistical analysis to discover the factors that influence patient satisfaction and the researchers’ methods was performed. Secondly, we executed a bibliometric analysis to find the most influential authors and documents within this theme.

The statistical analysis results yielded multiple determinants of satisfaction within diverse research areas, such as medicine, business, and the social sciences. Medical care, communication with the patient, and waiting time, patient’s age, perceived health status, and patient’s education are the factors that most influence patient satisfaction. Each one of these factors can create a positive or negative experience for the patient. Patient satisfaction directly connects to the loyalty of the patient towards the healthcare provider. Patient loyalty results in positive behaviors such as healthcare providers’ recommendations, compliance, and higher healthcare service usage, thus increasing profitability. With healthcare becoming an increasingly competitive market, measuring healthcare satisfaction and quality can help managers control, improve, and optimize several organizational aspects. While some markets and industries try to improve customer orientation, healthcare practitioners have to remain alert for the modifying behaviors of patient expectations [10,16,28,112]. An important market arising in the past few years has been that of medical tourism, and Ghasemi et al. [113] have comprehensively studied the impact of cost and quality management in patient satisfaction in this market. The authors verified the existence of a relationship of these three dimensions. Therefore, it becomes obvious that cost impacts satisfaction, a topic that has been overlooked by many researchers. Additionally, social media has been deemed as a relevant way for customers to express their satisfaction levels [114,115,116]. The influence of this route on the results should be better understood in future research.

The importance of the determinants of patient satisfaction can be assessed through several methods, as has been previously seen. Due to the ease of handling and computation, factor and regression analyses are the healthcare management methods. However, despite MUSA’s low usage rate, this is a useful method, with many advantages over the traditional customer satisfaction models. It considers the customers’ judgments in the way they are expressed in the questionnaires [25,67]. One must be careful when using ordinal data within models that are not suitable for the former; however, a lesson from our research is that some researchers are still struggling with those models despite the mathematical objections that these face. MUSA’s low usage in healthcare and its potential compared with other alternatives creates opportunities for broader dissemination of studies using that model. Other theoretically suitable alternatives should also be highlighted, including MUSA-INT, the integrated use of MUSA (or MUSA-INT) alongside benchmarking techniques (like DEA or the Benefit of Doubt), and some useful and appropriate multiobjective interval programming models.

Of course, the highlighted fact that patient satisfaction and health quality are not linearly related, and that the patient cannot always properly assess the provider’s performance, is a limitation that any satisfaction-based study faces. Despite the obvious need to study satisfaction determinants (and several reviews have tried to deal with this issue), the consistency of results is somehow absent. Perhaps the socio-economic differences among patients and even the characteristics of the health care systems in which they are part of may help to explain this lack of consistency. Future research should focus on this matter. Another possible limitation is the fact that we did not distinguish between inpatients and outpatients in this study. Although satisfaction may depend on the service itself, the same patient can be either an inpatient or an outpatient in different moments, meaning that the next experience (as an outpatient) will be somehow biased by the previous one (as an inpatient or vice-versa). It may help to justify the fact that often the term “patient satisfaction” seems more appropriate to study the healthcare facility as a whole, as it was one of the objectives in this study.

With regard to the second part of this study, an analysis of the literature through bibliographic methods featured the most important aspects of patient satisfaction research. Based on the article’s co-citation analysis, *Patient satisfaction: A review of issues and concepts* by Sitzia and Wood was the most relevant document. The published date of this article is 1997; thus, it was not included in our collection. However, when the reference list of the sample articles was analyzed, it was present on most of the lists. From the bibliographic coupling analysis, *Predictors of Patient Satisfaction* by Jackson et al. was the most critical document. This document was published in 2001, and thus it was included in our collection. These two articles are considered the most important and impactful in the area of patient satisfaction. The author’s co-citation and bibliographic coupling analysis revealed that Cleary and Otani are two of the most influential authors in this field. Both authors have collaborated in many articles in this research area; they are, thus, considered to be two of the most relevant researchers. Cleary’s articles related to patient satisfaction were published in the 1990s and are not included in our collection. Otani, however, is the author with the most documents in our collection. Despite the fact that his number of citations is not the highest, this author is influential because of the high number of collaborations in the field. From the journal’s co-citation and bibliographic coupling analysis, *Social Science & Medicine* and *Medical Care* were the most influential in patient satisfaction. However, considering general medicine/health, these journals were not the ones with the highest impact.

The knowledge obtained from this systematic review can be seen as an essential foundation for additional studies, and can be used to enhance further knowledge among healthcare practitioners, researchers, and scholars.

## Figures and Tables

**Table 1:** Review of collected articles.

Authors	Country of Study	Sample	Methodology	Quality Dimensions & Drivers	Dependent Variable	Main Factors Affecting Satisfaction
[12]	Belgium, United Kingdom, Finland, Germany, Greece, Ireland, Netherlands, Norway, Poland, Spain, Sweden, Switzerland and the USA;	61,168 surveys from nurses in 488 European hospitals and 617 USA hospitals, and 131,318 surveys from patients in 210 Europeans hospitals and 430 USA hospitals;	Logistic regression analysis; odds ratio (OR); robust logistic regression with cluster; *p*-value;	Nursing care; environment; burnout; dissatisfaction; intention to leave the job; patient safety; nursing care	Overall patient satisfaction with nursing care; Willingness to recommend hospital;	- nursing care; - environment;
[29]	21 European countries;	33,734 surveys from 21 European countries;	Additive ordinary least-squares regressions; *p*-value; r-square;	Service experience; fulfilment of expectations; perceived health status; patient’s personality;	Overall patient satisfaction;	- fulfilment of expectations; - provider type; - insurance;
[11]	USA	500 surveys from patients of 38 different physicians;	Kruskal–Wallis test; *p*-value; maximum likelihood ratio; chi-square; maximum-likelihood ordered logit models;	Fulfillment of expectations; information about symptoms duration; information about symptom resolution; patient’s age; patient’s autonomy;	Overall patient satisfaction;	- fulfillment of expectations; - information provided; - patient’s autonomy;
[30]	Bangladesh	1913 surveys from a public hospital;	Multivariate regression analysis; box plots; *p*-value;	Working hours; waiting time; medical care; doctor’s attitudes; appointment duration; privacy; physical examination; information provided; advice given by doctor;	Overall patient satisfaction;	- appointment duration; - privacy; - physical examination; - information provided; - the advice given by the doctor;
[31]	Scotland	2249 surveys from five public hospitals;	Spearmen correlation coefficient (SCC); *p*-value; multivariate linear regression; r-square;	Patient’s age; patient’s gender; health status; patient’s education; coordination of care; comfort; emotional support; respect for patient’s preferences; involvement of family; continuity of care;	Overall patient satisfaction;	- comfort; - emotional support; - respect for patient’s preferences;
[32]	Bangladesh	216 surveys from 57 hospitals and clinics;	Factor analysis; varimax rotation; Cronbach’s alpha; *p*-value; multiple regression analysis; r-square;	Ability to answer questions; doctor’s assurance; nurse’s assurance; staff’s assurance; communication with the patient; baksheesh; doctor’s attitudes;	Overall patient satisfaction;	- doctor’s attitudes; - doctor’s assurance; - nurse’s assurance; - staff’s assurance; - ability to answer questions; - communication with the patient;
[33]	South Africa	263 surveys from diabetic outpatients in two public hospitals;	Factor analysis; varimax rotation; Cronbach’s alpha; t-tests; *p*-value; PCC; analysis of variance (ANOVA); Kaiser-Meyer-Olkin (KMO);	Doctor’s kindness; doctor’s encouragement; doctor’s attitude; doctor’s ability to listen; doctors are supportive; doctor’s ability to answer questions; information provided; medical skills; information provided; maintenance of contact; follow up care; fair treatment; waiting time; availability of seat in waiting area; cleanliness; privacy;	Overall patient satisfaction;	- medical care; - doctor’s attitudes; - doctor’s kindness; - medical skills; - information provided; - doctor’s ability to answer questions; - cleanliness;
[34]	France	533 surveys from 12 medical services at a university hospital;	Bivariate and multivariate ordinal polychotomous analysis; *p*-value; t-tests; Pearson correlation coefficient (PCC); chi-square; OR;	Admission process; nursing care; medical care; information; hospital environment; overall quality of care; recommendations; patient’s age; patient’s gender; distance to the hospital; community size; patient’s BMI index; patient’s Karnofsky index; assistance needed at the hospital; patient’s autonomy; length of stay; attitude towards the length of stay; privacy;	Overall patient satisfaction;	- patient’s age; - perceived health status; - admission process; - patient’s autonomy; - privacy; - length of stay;
[35]	Turkey	369 surveys from one public hospital;	SERVQUAL; Cronbach’s alpha; *p*-value; average variance extracted; composite assurance; factor analysis; varimax rotation; structural equation modelling (SEM);	Accommodations; communication with the patient; empathy; skills; ability to answer questions;	Overall patient satisfaction;	- kindness; - skills;
[36]	USA	5232 surveys from the emergency department;	Cronbach’s alpha; SCC; *p*-value; ordinal regression model; OR; likelihood ratio;	Communication with family; waiting time; received help when needed; identification of health professionals; discharge process; information about return to the emergency department; signs of being aware regarding illness; side effects; provision of medication; takes medication as advised; results of medical exams; follow-up appointment; information provided; doctor’s attitudes;	Overall patient satisfaction; Willingness to return;	- doctor’s attitudes; - waiting time; - information provided; - results of medical exams;
[37]	USA	21,689 surveys from seven Veterans Affairs (VA) medical centres;	t-test; Wilcoxon rank-sum test; chi-square; multivariate linear regression; Cronbach’s alpha; r-square; *p*-value;	Patient’s age; patient’s gender; marital status; patient’s education; income; occupation; health status; received care outside VA; primary care visit in previous 12 months; distance from clinic; clinic site; provider type; provider’s gender; continuity of care;	Overall patient satisfaction;	- continuity of care;
[38]	Netherlands	66,611 surveys from 8 university hospitals and 14 general hospitals;	Multilevel analysis; intra-class correlation coefficient (ICC); chi-square;	Patient’s gender; patient’s age; patient’s education; health status; hospital type; hospital size; population density; admission process; nursing care; medical care; communication with the patient; patient autonomy; discharge process;	Overall patient satisfaction;	- patient’s age; - self-perceived health status; - patient’s education; - admission process; - nursing care; - medical care; - communication with the patient; - patient autonomy; - discharge process;
[39]	Italy	396 surveys from the dermatology department;	Principal components analysis (PCA); *p*-value; multiple logistic regression;	Patient’s age; patient’s gender; patient’s education level; region of residence; duration of disease; illness impact; quality of life, regarding emotions; quality of life, regarding symptoms; quality of life, regarding functioning; medical care; the accuracy of dermatological visit; doctor’s ability to listen; concern for questions; appointment duration; information provided;	Overall patient satisfaction;	- information provided; - doctor’s attitudes; - patient’s age; - illness impact;
[40]	USA	437 surveys from the emergency department of a municipal hospital;	t-tests; chi-square; Mann-Whitney U tests; *p*-value; univariate and multivariate analysis;	Patient’s age; patient’s gender; patient’s race; insurance; priority code; visit-time of the day; day of the week; disposition; reception courtesy; reception helpfulness; privacy; nursing care; information about treatment provided by nurses; nurses’ skills; information about condition provided by doctors; medical exams explanation provided by doctors; next steps explained by doctors; follow-up instructions; discharge instructions; X-ray staff courtesy; staff care; communication with the family;	Overall patient satisfaction; Willingness to recommend hospital;	- staff care; - safety; - follow-up instructions; - nurse’s skills; - waiting time; - patient’s age (solely for willingness to recommend hospital); - insurance (solely for willingness to recommend hospital);
[41]	United Kingdom	1816 surveys from the oncology department;	PCA; varimax rotation; Mann-Whitney U test; Kruskal-Wallis; ANOVA; Bonferroni coefficient; *p*-value;	Patient’s age; physician’s age; patient’s gender; physician’s gender; patient’s physiological morbidity; waiting time; tumor site; type of treatment;	Overall patient satisfaction;	- waiting time; - patient’s age; - the patient’s physiological morbidity;
[42]	Sweden	7245 surveys;	Chi-square; PCC; Fisher’s exact probability test;	Patient’s age; patient’s gender; self-perceived health status; the origin of birth; patient’s education; living area; living condition; fulfillment of expectations; medical care; waiting time; patients’ participation in making decisions about treatment;	Overall patient satisfaction;	- patient’s age; - patient’s education; - self-perceived health status; - patient’s nationality; - fulfillment of expectations; - medical care; - waiting time; - patients’ participation in making decisions about treatment;
[43]	Norway	10,912 surveys from 63 hospitals;	Test-retest assurance; ICC; Cronbach’s alpha; PCC; multivariate linear regression analysis; multilevel linear regression analysis; *p*-value;	Fulfillment of expectations; nursing care; medical care; incorrect treatment; health personnel in general; organization; waiting time; pain relief; communication with the patient; next of kind–handling; medical equipment; patient demographics;	Overall patient satisfaction;	- nursing care; - fulfillment of expectations; - medical care; - perceived incorrect treatment;
[44]	USA	1868 surveys from private outpatient physical therapy clinics;	Inter-item correlation; *p*-value; multiple regression analysis; r-square; Cronbach’s alpha; chi-square; PCA; oblimin rotation;	Therapist’s ability to answer questions; therapist’s ability to listen; therapist’s kindness; appointment duration; information provided; staff’s kindness; cleanliness; medical equipment; working hours; the complexity of registration; waiting area; parking; waiting time; location;	Overall patient satisfaction;	- appointment duration; - information provided;
[26]	Germany	8428 surveys from 39 hospitals;	PCA; Cronbach’s alpha; non-parametric Kruskal–Wallis test; *p*-value; chi-square; Fisher’s exact test; logistic regression analysis;	Fulfillment of expectations; outcome; the kindness of the nurses; the kindness of the doctors; organization of procedures and operations; quality of food; accommodation; medical care; discharge process; physician’s knowledge of patient anamnesis; admission process; communication with the patient; cleanliness;	Overall patient satisfaction;	- outcome; - the kindness of nurses; - the kindness of doctors; - the organisation of procedures and operations; - quality of food; - accommodations; - medical care; - discharge process; - physician’s knowledge of patient anamnesis; - admission process;

**Table 2:** Statistical measures applied to all collected data.

	Sample Size	No. Methods	No. Criteria	No. Explanatory Variables	No. Critical Factors	No. Citations
Mean	18,640	1	8	3	4	50
Median	728	1	6	3	4	14
Mode	200	1	6	0	3	0
Standard Deviation	84,507	0.39	6	3	2	110
Coefficient of Variation	453%	33%	73%	100%	59%	222%
Minimum	37	1	0	0	1	0
Maximum	934,800	2	26	14	13	763

**Table 3:** Statistical measures applied to the articles with more than 100 citations.

	Sample Size	No. Methods	No. Criteria	No. Explanatory Variables	No. Critical Factors	No. Citations
Mean	18,784	1	8	4	5	278
Median	1913	1	6	2	4	243
Mode	N/A	1	4	0	3	126
Standard Deviation	43,937	0.49	5	4	3	190
Coefficient of Variation	234%	35%	63%	100%	56%	69%
Minimum	216	1	1	0	1	101
Maximum	192,486	2	16	13	10	763

**Table 4:** Ten of the most utilized journals. The citations presented in this table are the sum of the total citations of the articles published by each journal.

Journal	Articles	Citations
International Journal of Health Care Quality Assurance	8	178
International Journal of Environmental Research and Public Health	5	37
Social Science & Medicine	5	1274
International Journal for Quality in Health Care	4	327
Journal of Healthcare Management	4	199
Patient Preference and Adherence	4	38
BMC Health Services Research	3	76
PLOS One	3	36
Annals of Emergency Medicine	2	281
BMC Family Practice	2	30
BMC Research Notes	2	33
Health and Place	2	30
Health Expectations	2	12
Health Policy	2	71
Health Services Management Research	2	32

**Table 5:** Ten most studied countries from collected articles.

Country	Articles	Percentage (%)
USA	46	30%
Germany	13	8%
China	8	5%
Portugal	8	5%
Turkey	6	4%
United Kingdom	5	3%
Australia	5	3%
Pakistan	5	3%
Spain	5	3%
Italy	4	3%
Iran	4	3%

**Table 6:** Total citations (TC) and TC/year of the fifteen most cited publications.

Publication	Title	TC	TC/Year
[12]	Patient safety, satisfaction, and quality of hospital care: Cross sectional surveys of nurses and patients in 12 countries in Europe and the United States	763	8489
[11]	Predictors of patient satisfaction	522	2610
[31]	Patients’ experiences and satisfaction with health care: Results of a questionnaire study of specific aspects of care	333	1550
[32]	Service quality perceptions and patient satisfaction: a study of hospitals in a developing country	310	1753
[34]	Factors determining inpatient satisfaction with care	257	1353
[36]	Determinants of patient satisfaction and willingness to return with emergency care	243	1157
[37]	Continuity of care and other determinants of patient satisfaction with primary care	182	1138
[38]	Patient satisfaction revisited: A multilevel approach	153	1275
[30]	Client satisfaction and quality of health care in rural Bangladesh	145	725
[39]	Factors associated with patient satisfaction with care among dermatological outpatients	138	690
[40]	Determinants of patient satisfaction in a large, municipal ED: The role of demographic variables, visit characteristics, and patient perceptions	126	700
[41]	Factors affecting patient and clinician satisfaction with the clinical consultation: can communication skills training for clinicians improve satisfaction?	126	600
[42]	Patient characteristics and quality dimensions related to patient satisfaction	122	1109
[43]	Overall patient satisfaction with hospitals: Effects of patient-reported experiences and fulfilment of expectations	112	1244
[44]	Patient satisfaction with outpatient physical therapy: Instrument validation	106	558

## Data Availability

Not applicable.
